# Adaptive Bitline Voltage Countermeasure for Neighbor Wordline Interference in 3D NAND Flash Memory-Based Sensors

**DOI:** 10.3390/s23063212

**Published:** 2023-03-17

**Authors:** Hanshui Fan, Xuan Tian, Huiting Peng, Yinfeng Shen, Liang Li, Ming Li, Liming Gao

**Affiliations:** 1 Institute of Microelectronic Material & Technology, School of Material Science and Engineering, Shanghai Jiao Tong University, Shanghai 200240, China; 2 SanDisk Information Technology Co., Ltd., Shanghai 200241, China

**Keywords:** 3D NAND flash memory, bitline voltage, channel potential, DIBL effect, device model, MONOS, neighbor wordline interference, read bias, TCAD

## Abstract

Three-dimensional NAND flash memory is widely used in sensor systems as an advanced storage medium that ensures system stability through fast data access. However, in flash memory, as the number of cell bits increases and the process pitch keeps scaling, the data disturbance becomes more serious, especially for neighbor wordline interference (NWI), which leads to a deterioration of data storage reliability. Thus, a physical device model was constructed to investigate the NWI mechanism and evaluate critical device factors for this long-standing and intractable problem. As simulated by TCAD, the change in channel potential under read bias conditions presents good consistency with the actual NWI performance. Using this model, NWI generation can be accurately described through the combination of potential superposition and a local drain-induced barrier lowering (DIBL) effect. This suggests that a higher bitline voltage ($V_{bl}$) transmitted by the channel potential can restore the local DIBL effect, which is ever weakened by NWI. Furthermore, an adaptive $V_{bl}$ countermeasure is proposed for 3D NAND memory arrays, which can significantly minimize the NWI of triple-level cells (TLC) in all state combinations. The device model and the adaptive $V_{bl}$ scheme were successfully verified by TCAD and 3D NAND chip tests. This study introduces a new physical model for NWI-related problems in 3D NAND flash, while providing a feasible and promising voltage scheme as a countermeasure to optimize data reliability.

## 1. Introduction

Due to the characteristics of high storage density and low bit cost, 3D NAND flash has become the mainstream structure of high-performance memory chips [1,2]. Meanwhile, sensor systems are becoming increasingly complex in scale and function, and it is critical to be able to collect, store, and manage large amounts of data. The nodes of sensor networks are capable of sensing physical quantities such as temperature, humidity, and pressure in the environment. These data, after being processed and stored, can be used to monitor and control various applications such as smart homes, healthcare, industrial automation, and more [3]. Therefore, ensuring complete and accurate access to stored data is critical to the stable operation of sensor systems. NAND flash memory, as a non-volatile memory device, is the most widely used data storage method in modern sensor systems due to its fast access [4,5]. At the same time, NAND flash memory has high endurance and shock resistance, which makes it possible to store data stably for a long time under extreme operating conditions [6]. In addition, advanced sensor systems, such as wireless sensor networks (WSNs), use an approach whereby they sense, store, merge, and send to monitoring [7]. Sensors store generated data in flash memory cells and perform in-network calculations when required [8,9,10]. Advanced flash memory technology provides sensor networks with powerful capabilities for the collection, storage, and processing of data.

To meet the demand for large data capacity growth, 3D NAND flash keeps scaling and storage layers are constantly stacked [11,12]. As a result, some reliability problems, such as data disturbance and retention, become more serious and neighbor wordline interference (NWI) is one of these problems [13,14]. NWI refers to the effect wherein the programmed cell causes the threshold voltage ($V_{t}$) of its neighbor cells to shift [15]. The $V_{t}$ shift can result in an incorrect state read from the victim cell [16]. NWI has long existed in high-density flash memory and is difficult to completely eliminate. Different from floating gate-based 2D NAND, due to changes such as 3D vertical architecture, poly-silicon channels, and charge traps, the NWI effect in 3D NAND needs further investigation [17,18,19].

Previous studies on 3D NAND NWI were undertaken mainly from the perspective of trapped electrons, suggesting that the charge trap layer (CTL) between adjacent wordlines (WLs) was programmed into redundant electrons, shifting the $V_{t}$ of neighbor cells [20]. Therefore, some studies adopted methods such as asymmetric $V_{pass}$ [21] and the CTL blocking process [22] to eliminate the NWI that resulted from the electron reduction between WLs. Unfortunately, as the process size of WL and isolation becomes smaller, these redundant electrons become hard to avoid and eliminate. Furthermore, it is almost impossible to accurately define and measure the influence scopes of trapped electrons. In fact, NWI interference comes from potential interference. Based on the channel potential continuity theory in 3D NAND structure, the trapped electrons of other flash cells can affect the channel potential of the victim cell and then interfere with its read $V_{t}$ [23]. From the perspective of channel potential, the NWI can be investigated more intuitively and accurately. In this way, some studies reduced NWI by enhancing the $V_{read}$ applied to neighbor cells [24] or by adopting reverse order program [25].

However, the NWI, especially in 3D NAND, is affected by many electronic factors, and there is no unified approach to deal with various ramifications of NWI. Research has shown that the electrons between the WL space contribute about 40% of NWI [26], so there remain other disturbing factors. In fact, in 3D NAND, the device operating parameters of read and write will also affect NWI, and it should be accurately integrated in a physical model. Moreover, the NWI varies greatly under different combinations of cell states, inevitably resulting in various degrees of residual NWI.

Therefore, in this paper, a new 3D NAND device model combined with channel potential was established to further investigate the NWI mechanism. It integrates the effects of various device parameters, such as $V_{read}$, $V_{bl}$, $I_{sense}$, and different $V_{t}$ on NWI. An NWI analysis scheme by channel potential is proposed to optimize the original method by trapped electron. Using technology computer-aided design (TCAD) simulation, a 3D NAND structure model with eight-layer WLs was built. The physical device model indicated that the NWI effect is closely related to device operating bias. TCAD simulations also confirmed this and found that the applied device bias can result in a significant difference in channel potential changes caused by NWI. Thus, the theory of channel potential superposition and the local drain-induced barrier lowering (DIBL) effect were utilized to describe the mechanism of NWI generation, providing an efficient way to quickly analyze NWI problems from a device perspective. On this basis, an adaptive bitline voltage ($V_{bl}$) countermeasure for 3D NAND arrays was proposed to reduce the NWI on triple-level cells (TLC) cells rationally and maximally. Finally, these proposed theories and methods were successfully verified by TCAD experiments and 3D NAND chip tests. This $V_{bl}$ scheme in 3D NAND provides a flexible and extensible solution to alleviate NWI, promising to efficiently enhance data reliability.

## 2. Simulation Set-Up for NWI

**Figure 1:**
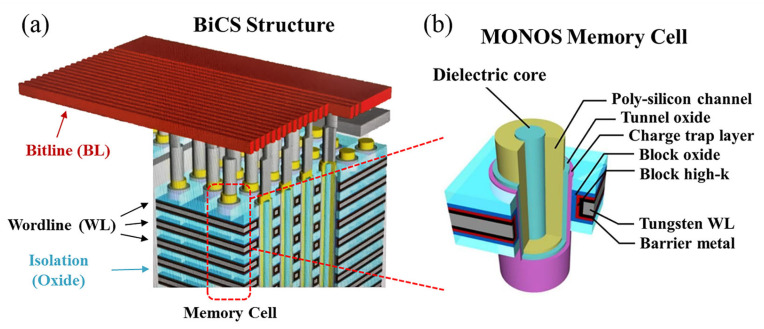
(**a**) Three-dimensional schematic for part of the bit cost scalable (BiCS) architecture in 3D NAND flash. (**b**) Detailed multilayer structure of a single MONOS memory cell.

**Figure 2:**
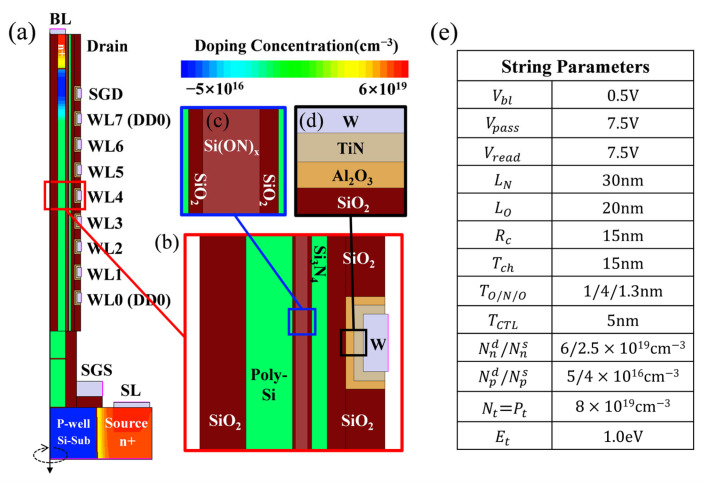
(**a**) Schematic cross section of a 3D NAND bitline string used in TCAD simulation. N-type and p-type doping concentrations are shown as color levels. Enlarged images of (**b**) MONOS memory cell, (**c**) tunnel oxide layer, and (**d**) block layer. (**e**) Main bitline string parameters.

Figure 1a shows the bit cost scalable (BiCS) architecture of a 3D NAND flash, where wordline (WL) and isolation are alternately stacked to form multiple layers for storage. A large number of memory holes vertically cross the layers and form individual memory cells. Figure 1b illustrates the structure of a metal-oxide–nitride-oxide–silicon (MONOS) memory cell used in the TCAD simulation. From outside to inside, it consists of a tungsten WL, block layer (BLK), charge trap layer (CTL), tunnel oxide layer (TNL), poly-silicon channel, and dielectric core. The BLK consists of barrier metal/block high-k/block oxide and the TNL consists of oxide/nitride/oxide (O/N/O), both of which are three-layer structures. Figure 2a shows the TCAD modeling structure of a single 3D NAND bitline (BL) string, and a single MONOS memory cell in the red box. The device doping concentrations are shown as color levels.

Figure 2b–d shows the composition and material details of a MONOS cell, a TNL, and a BLK respectively. The simulation assumes rotational symmetry along the vertical central axis of the memory hole, so that the cylindrical mathematical method can be used to simulate a 3D cylindrical device with a 2D mesh. The string parameters of the TCAD model are shown in Figure 2e. $L_{N}$ and $L_{o}$ are ON pitch length, and $R_{c}$ is radius of core. $T_{ch}$, $T_{O/N/O}$, and $T_{CTL}$ are thickness of channel, O/N/O, and CTL, respectively. $N_{n}^{d}$/$N_{n}^{s}$ and $N_{p}^{d}$/$N_{p}^{s}$ are n-type and p-type doping concentrations of drain and source, respectively. $N_{t}$ and $P_{t}$ are electron and hole trap density respectively, and $E_{t}$ is electron trap energy in CTL [27]. 

Three-dimensional NAND cells are programmed forward from sourceline (SL) to bitline (BL) (low WL1 to high WL6). WL0 and WL7 serve as dummy WLs that do not participate in the program, designed to mitigate the potential difference between SGS/SGD and WLs. The simulation uses an incremental single-pulse program voltage $V_{PGM}$, of which the high-period increases from $V_{PGM,\;initial}$ to $V_{PGM,\;target}$. The NAND test program is executed with the incremental step pulse program (ISPP) voltage $V_{PGM,pulse}$. The cell for simulation and test is of the TLC type, and each cell contains eight states (three bits/cell). Different program times $t_{PGM}$ can ensure that the memory cell is programmed to the specific $V_{t}$ according to the required state. The bitline voltage $V_{bl}$ is 0.5V in both program and read, where $V_{pass}$ is also equal to $V_{read}$. The $V_{t}$ value is read according to the WL gate voltage when $I_{cell}$ is 10nA, which can be obtained from the $I_{d}$-$V_{g}$ curve in simulation. The NWI effect on WL4 caused by WL5 was simulated. The difference of WL4 read $V_{t}$ before and after WL5 program is the $V_{t}$ shift of WL4 caused by NWI, which will be explained next.

## 3. Physical Device Model of NWI

The NWI effect is caused by the programming of neighbor flash cells, resulting in $V_{t}$ interference to the victim cell. In practice, NWI is calculated as $V_{t}$(shift) = $V_{t}$(NWI) − $V_{t}$(initial). This is the common approach used to measure the NWI value in both TCAD simulation and 3D NAND chip test. On the process, first program WLn−1, and then program WLn. At this time, read the $V_{t}$ of WLn as $V_{t}$(initial). On this basis, program WLn+1 and finally read the $V_{t}$ of victim WLn as $V_{t}$(NWI).

In this study, an NWI model of a 3D NAND device was established according to the NWI generation environment and the theoretical expression at the device level was derived. In the linear and saturation regions of the standard $I_{d}$-$V_{ds}$ curve, the drain current $I_{d}$ of a flash cell can be modeled by the gate-source voltage $V_{gs}$ and drain-source voltage $V_{ds}$ as:

(1) $$I_{d}=\frac{\mu_{n}C_{ox}W}{2L}\left[2\left(V_{gs}-V_{t}\right)V_{ds}-V_{ds}^{2}\right]$$

where $\mu_{n}$ is the charge-carrier effective mobility,$\;C_{ox}$ is the gate oxide capacitance per unit area, *W* is the gate width, and *L* is the gate length.

**Figure 3:**
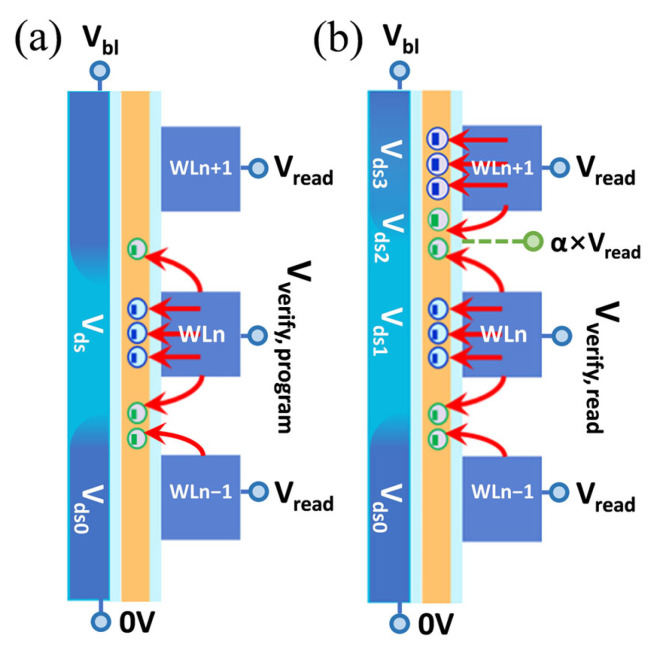
Schematic boundary conditions of operation bias applied to the victim WLn and neighbor WLs (**a**) before WLn+1 is programmed and (**b**) after WLn+1 is programmed. The channel potential variations of each cell are also reflected.

When WLn is read for the first time, the bias voltage applied to the device is shown in Figure 3a. It is worth mentioning that the actual 3D NAND device performs read operations under the all bit line (ABL) sense method [28]. As the cell current of the sense amplifier circuit reaches $I_{sense}$, the $V_{gs}$ of the read cell is approximately equal to or slightly larger than the cell $V_{t}$. Thus, the read cell is in the saturation region of the $I_{d}$-$V_{ds}$ curve, and the ($I_{sense}$, $V_{gs}$) point falls near the advance pinch-off point. At this point, there is:

(2) $$V_{ds}=V_{gs}-V_{t}$$

According to (2), the drain current is modeled as (3). Thus, in this case, the sense current in the ABL sense is obtained as (4):

(3) $$I_{d}=\frac{\mu_{n}C_{ox}W}{2L}{\left(V_{gs}-V_{t}\right)}^{2}$$

(4) $$I_{sense}=K_{n}{\left[V_{verify,\;\;PGM}-V_{ds0}-\left(V_{t0}-DIBL\times V_{ds}\right)\right]}^{2}$$

where

(5) $$K_{n}=\frac{\mu_{n}C_{ox}W}{2L}$$

(6) $$V_{ds}=V_{bl}-V_{ds0}$$

The value of DIBL can be calculated as (7), where DIBL is a constant for specific device materials and structures. It is controlled by the characteristic length L as (8), based on the short channel effect model:

(7) $$DIBL=-\frac{V_{t}-V_{t}^{low}}{V_{bl}-V_{bl}^{low}}$$

(8) $$L=\sqrt{\frac{\varepsilon_{Si}\times EOT\times T_{Si}}{\varepsilon_{OX}}}$$

where  EOT is the equivalent oxide thickness of the BLK, CTL, and TNL gate stacks, $T_{Si}$ is the thickness of channel, and $\varepsilon_{Si}$ and $\varepsilon_{OX}$ are the dielectric constants of Si and gate oxide.

After programming WLn+1, we read WLn a second time. The bias voltage applied to the device is shown in Figure 3b. In this case, the WLn cell is still in the saturation region of the $I_{d}$-$V_{ds}$ curve. The gate voltage is $V_{verify,read}$ and the drain-source voltage is now reduced to $V_{ds1}$. Therefore, $I_{sense}$ can be written as:

(9) $$I_{sense}=K_{n}{\left[V_{verify,\;\;read}-V_{ds0}-\left(V_{t0}-DIBL\times V_{ds1}\right)\right]}^{2}$$

(10) $$V_{ds1}=V_{bl}-V_{ds3}-V_{ds2}-V_{ds0}$$

The newly trapped electrons of WLn+1 enhance the local negative potential. Due to the same $V_{read}$, a channel potential drop $V_{ds3}$ is actually generated. The WLn+1 cell is in the linear region with a small $V_{ds3}$. Therefore, the $I_{sense}$ of WLn+1 can be written as (11). The WLn+1 channel is in the pass state under $V_{read}$, so the potential drop $V_{ds3}$ is small and the $V_{ds3}^{2}$ can be ignored as (12):

(11) $$I_{sense}=K_{n}\left[2\left(V_{read}-V_{bl}-V_{t,n+1}\right)V_{ds3}+V_{ds3}^{2}\right]$$

(12) $$I_{sense}=2K_{n}\left(V_{read}-V_{bl}-V_{t,n+1}\right)V_{ds3}$$

All the newly trapped electrons between WLn and WLn+1 can be regarded as a parasitic cell, which will affect the $V_{t}$ of WLn. Since the channel potential drop ($V_{ds2}$) of the parasitic cell is also very small, it is in the linear region of the $I_{d}$-$V_{ds}$ curve. Therefore, the $V_{ds2}^{2}$ and $2V_{ds2}V_{ds3}$ in (13) can be ignored. The channel conduction parameter of the parasitic cell is considered to be $K_{n}^{\prime }$ similar to $K_{n}$, so $I_{sense}$ of the parasitic cell is derived as (14):

(13) $$I_{sense}=K_{n}^{\prime }\left[\begin{array}{c} 2\left(\alpha \times V_{read}-V_{bl}-V_{t,parasitic}\right)V_{ds2}\\ +V_{ds2}^{2}+2V_{ds2}V_{ds3} \end{array}\right]$$

(14) $$I_{sense}=2K_{n}^{\prime }\left(\alpha \times V_{read}-V_{bl}-V_{t,parasitic}\right)V_{ds2}$$

After solving the equations, including (4), (6), (9), (10), (12), and (14), the $V_{t}$ change of WLn between two read operations is obtained. Namely, the NWI measurement result is obtained as follows:

(15) $$V_{t,NWI}=V_{verify,read}-V_{verify,PGM}$$

(16) $$V_{t,NWI}=DIBL\times \left[\begin{array}{c} \frac{I_{sense}}{2K_{n}^{\prime }\left(\alpha \times V_{read}-V_{bl}-V_{t,parasitic}\right)}\\ +\frac{I_{sense}}{2K_{n}\left(V_{read}-V_{bl}-V_{t,n+1}\right)} \end{array}\right]$$

The proposed theoretical device model in (16) shows the main factors leading to the NWI effect, including $I_{sense}$, $V_{read}$, $V_{bl}$, $V_{t,parasitic}$ and $V_{t,n+1}$. These factors are adjustable parameters that are worth investigating for NWI problems. Briefly, according to these factors, the NWI value can be reduced by increasing $V_{read}$, decreasing the amount of parasitic cell electrons, decreasing $I_{sense}$ ($I_{sense}$ reflects the program level of WLn), decreasing $V_{t,n+1}$ (lower program state of WLn+1), increasing channel length (weakening DIBL effect), and so on. These provide valuable theoretical guidance and mitigation schemes for the subsequent research. Moreover, there is the question of how exactly these factors work and induce NWI during device operation. To explore this question, the channel potential mechanism for the NWI effect is further investigated in the next section.

## 4. Channel Potential Simulation for NWI

### 4.1. Boundary Conditions with Device Read Bias

NWI is essentially a channel potential disturbance controlled by the channel potential continuity theory. The trapped electrons of WLn+1 appear to be the direct cause, whereas the channel potential change under the victim cell is the intrinsic cause. Programming WLn+1 causes a local channel potential drop near WLn, resulting in a higher read $V_{t}$ of WLn.

**Figure 4:**
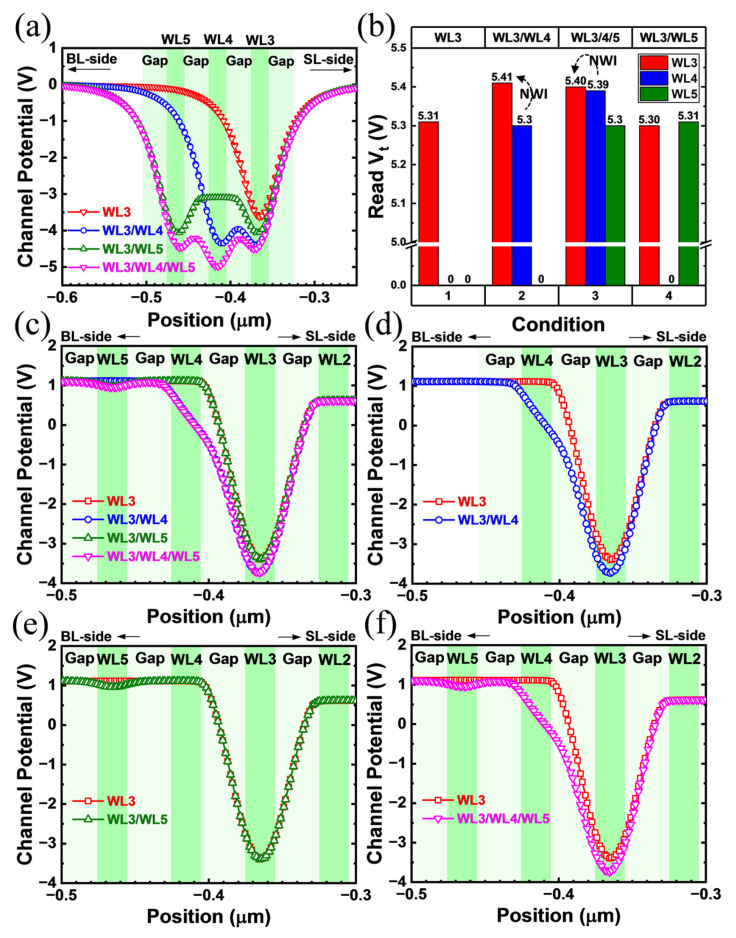
(**a**) Channel potential distribution curves in floating mode without boundary bias under four programming sequences. (**b**) The $V_{t}$ read from each WL cell in four programming sequences. Zero represents a WL that is not programmed. (**c**) Channel potential distribution curves with read bias under four programming sequences (read WL3). Isolate overlapping curves, (**d**–**f**) respectively show the channel potential interference on the WL3 after programming WL4, WL5, and WL4/WL5.

TCAD simulation results show that the channel potential of the victim cell varies greatly under different device bias conditions. WL3/WL4 means program WL3 with program WL4 following. The channel surface potential near the TNL side is used to reflect the changes. The channel potential distribution of four programming sequences, with no bias applied to this string, is shown in Figure 4a. Focusing on WL3, its channel potential suffers from varying degrees of potential superposition related to WL distance. This indicates that either WLn+2 programming or WLn+1 programming can produce a large increase in the static channel potential barrier of WLn, and that a closer WL brings a greater impact. Moreover, in the WL3/4/5 sequence, WL4 is affected by the closest WLs on the left and right, so its electrostatic potential is the lowest.

However, in Figure 4b, the actual NWI test result shows that only WLn+1 programming has a significant NWI effect on WLn. WLn+2 programming has little effect on the $V_{t}$ of WLn. The height of the channel potential barrier does not reflect the size of its read $V_{t}$. This is inconsistent with the mutual effect of the channel potential situation as shown in Figure 4a. In fact, based on the TCAD results in Figure 4c, it was found that the bias condition is also essential for channel potential analysis in NWI. In Figure 4c, under the read bias conditions of $V_{bl}$, $V_{read}$ and $V_{verify}$, the channel potential distribution of each cell starts to present good consistency with actual NWI variations. Isolating these curves, it can be clearly seen in Figure 4d,f that WL4 raises the channel read potential barrier of WL3. Furthermore, the results in Figure 4e,f prove that WL5 only affects itself but does not affect WL3 on the channel potential. Thus, in read bias mode, only WLn+1 programming has a significant effect on the channel potential of WLn, whereas WLn+2 programming has almost no effect on it. Obviously, the pass voltage $V_{read}$ can significantly restore the channel potential of WLn+2, but it cannot offset the potential disturbance caused by the WLn+1 program while reading WLn.

This study visually shows the channel potential variation of the victim cell and the adjacent cells affected by NWI. It also illustrates that the analysis of NWI based on channel potential must be developed with the device operating mode and bias condition, otherwise there will be a large error in the results. In the next section, the sources of potential disturbance and the ways in which potential changes affect the read $V_{t}$ are explored. Meanwhile, the mechanism of NWI with the read bias and the NWI model derived above will be revisited through the channel potential theory.

### 4.2. Channel Potential Superposition and DIBL Effect

**Figure 5:**
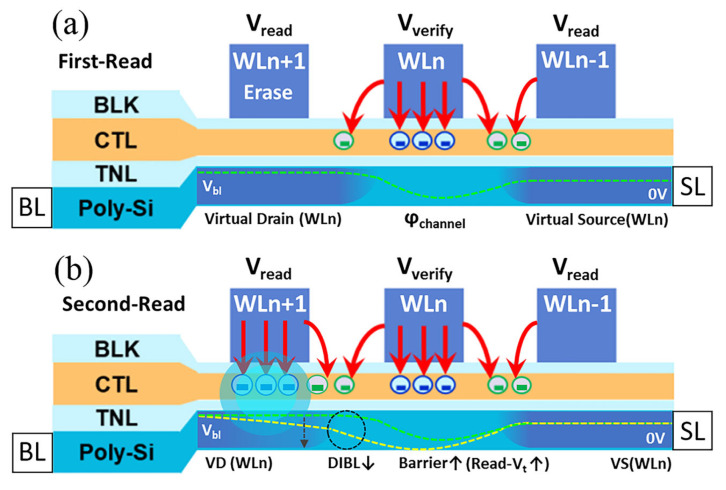
Schematic diagram of channel surface potential distribution near TNL side, when reading for (**a**) the first time and (**b**) the second time. The dashed line describes the distribution change of channel surface potential along the SL to BL direction.

The essence of reading a NAND cell is to judge whether the channel is on according to the channel current under gate verify voltages, and then obtain the $V_{t}$. The movement of electrons in the channel is driven by the electric potential difference from drain to source and is also affected by the local channel potential change due to the bias press. As shown in Figure 5a,b, the only difference between two read processes is that the WLn+1 cell is first in an erase state, but in (b) is programmed with a certain number of electrons. The WLn+1 channel region near the WLn side appears as an obvious potential disturbance, which is the potential cause of $V_{t}$(shift).

How does the regional channel potential change between these two read processes? After programming WLn+1, as shown in Figure 5b, a large number of electrons tunnel into the CTL where electrons are trapped. Due to the high density of trapped electrons in the CTL, a strong negative potential region is formed locally around the structure. The same $V_{read}$ was applied to WLn+1 as planned during both reads. On the second read, under the superposition of the strong negative electric potential, $V_{read}$ was insufficient to raise the channel potential to the original level as the first read. Therefore, the channel potential of WLn+1 decreases significantly, which will affect the next reading process of WLn.

Unlike the structure of 2D NAND, there is no fixed doping region between the memory cells in 3D NAND where a polysilicon column can be utilized as a channel through all the cells [29]. Therefore, in 3D NAND, to read the $V_{t}$ of WLn, the pass voltage $V_{read}$ must first be applied to the other WLs to transfer the electrons and potential from source and drain (at each end of the NAND string) to the WLn channel. After that, the read verify process is performed on the target WLn, the channel potential of which will be significantly lower than that of the neighbor WLs with the $V_{read}$ applied.

In this case, the local channel near WLn+1 and WLn−1 correspond to the virtual drain (VD) and virtual source (VS) of cell WLn, respectively. The drain voltage $V_{bl}$ can be transferred to WLn+1 through the channel opened by $V_{read}$. However, as shown by the two dashed lines in Figure 5b, the superposition of strong negative electric potential under WLn+1 leads directly to the transferred $V_{bl}$ loss, with a lower potential line in the WLn+1 channel. As a result, the virtual drain potential of the WLn decreases. This is equivalent to a drain voltage drop when reading a single NAND cell. In the short-channel of the WLn cell, the virtual drain voltage decreases, and the channel potential barrier rises as a consequence of the drain-induced barrier lowering (DIBL) effect, with the read $V_{t}$ of WLn increasing as a result of NWI. This effect occurs in the channel region between WLn and WLn+1 and is considered a local DIBL effect.

**Figure 6:**
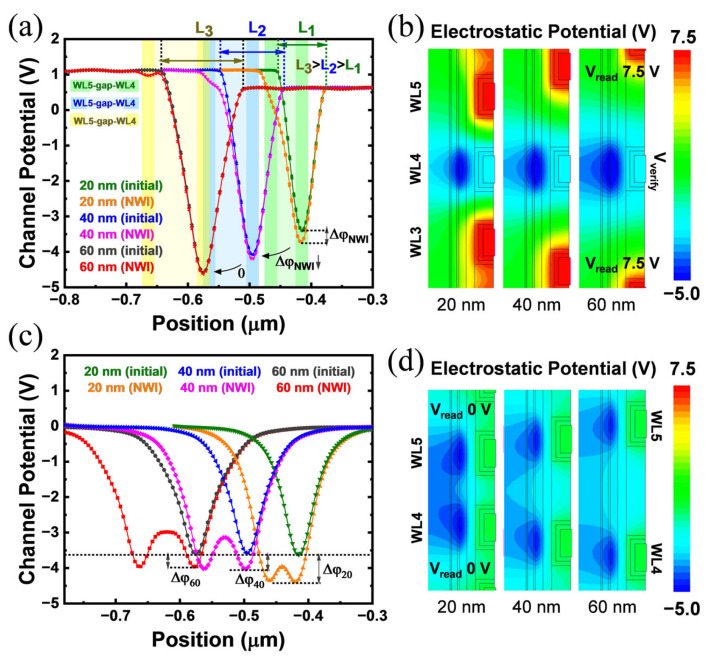
Channel potential distribution of the 3D NAND string with 20 nm, 40 nm and 60 nm WL gaps (isolations). (**a**) Simulation with read bias applied on boundary. (**c**) Simulation without any boundary bias. Both of the right patterns (**b**,**d**) visually show the electrostatic potential distribution around the victim WL, corresponding to the left.

For a deeper discussion, the channel potential superposition is the trigger for NWI. The programming of WLn+1 inevitably leads to the negative potential superposition from the CTL region to the channel region. In Figure 6a,b, the channel potentials of programmed WLn+1 are both shown to decrease significantly. As the first step leading to NWI, the channel potential superposition occurs once electrons are trapped as data storage. This causes the virtual drain potential of WLn to decrease and then induces the local DIBL effect.

The local DIBL effect is the root cause of NWI. The DIBL effect is a short channel effect that occurs when the channel length is smaller than the DIBL characteristic length L. As shown in Table 1, the TCAD experiment shows that when the WL gap increases, the NWI ($V_{t}$ (shift)) value decreases accordingly. When the WL gap is larger than about 40 nm, NWI is almost close to 0. In Figure 6a, when reading WLn, the channel lengths L_1_, L_2_, and L_3_ increase with larger WL gaps. At L_3_, the interference $\;\Delta \varphi_{\mathrm{NWI}}$ with the potential barrier tends to zero, as does the variation in Table 1. The longer channel length can weaken the local DIBL effect, eliminate the rise of the read potential barrier, and degrade NWI. Furthermore, in Figure 6b, the static channel potential superposition is investigated. This shows that the intrinsic potential interference still exists in the channel at larger gaps, although with a decreasing trend. However, different from Figure 6a, no obvious NWI is observed between the 40 nm to 60 nm gaps. In conclusion, it is the local DIBL effect that determines whether NWI is generated more than the potential superposition. Once the reading channel length exceeds the characteristic length L of DIBL, the local DIBL effect is no longer valid. In this case, even if the WLn+1 potential superposition still exists on the WLn virtual drain, it will not cause the raise of the WLn reading potential barrier, leading to an accurate read $V_{t}$ without NWI.

The combination of channel potential superposition and local DIBL effect principle can help to quickly analyze NWI in 3D NAND device. It is an extensible physical mechanism behind the device parameters $V_{read}$, $V_{bl}$, $V_{verify}$, and so on. Meanwhile, the proposed theory can guide a solution of the analogous NWI problems from more perspectives, such as channel potential enhancement or DIBL effect suppression.

## 5. Adaptive Bitline Voltage Countermeasure

### 5.1. Mechanism and Simulation of Adaptive V_bl_

Based on the proposed model and the potential theory, it is obvious that $V_{bl}$ is an important parameter affecting NWI. The variation of $V_{bl}$ will be transferred to the read region of the target cell through the passed channel, and then change its drain potential. Therefore, increasing the $V_{bl}$ that is applied to the bitline can also feasibly boost the virtual drain voltage of the WLn cell. If the bitline voltage is increased from $V_{bl}$ to $V_{blc}$ on the second read to properly compensate the drain potential loss, the NWI effect on the WLn cell can be whittled down.

In fact, in terms of the proposed device-level NWI model, this scheme can be further proved and derived as:

(17) $$\Delta V_{bl}=V_{blc}-V_{bl}$$

(18) $$V_{t,NWI}=DIBL\times \left[\begin{array}{c} \frac{I_{sense}}{2K_{n}^{\prime }\left(\alpha \times V_{read}-V_{bl}-V_{t,parasitic}\right)}\\ +\frac{I_{sense}}{2K_{n}\left(V_{read}-V_{bl}-V_{t,n+1}\right)}-\Delta V_{bl} \end{array}\right]$$

**Figure 7:**
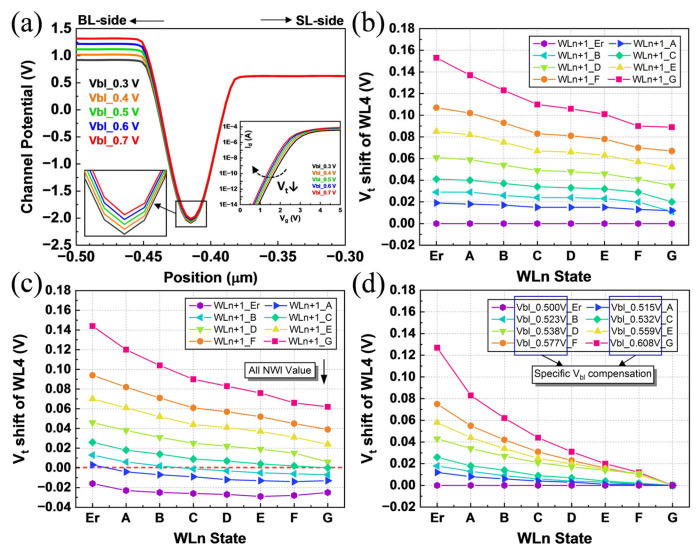
(**a**) The channel potential barrier and *I_d_*-*V_g_* curve of the victim cell when reading with different *V_bl_* applied on BL. (**b**) The NWI curves for all combinations of WLn and WLn+1 states. One curve represents the NWI of WLn states affected by a certain WLn+1 state. (**c**) The all-state NWI curves downward as *V_bl_* increases from 0.50 V to 0.53 V. (**d**) The all-state NWI curves are optimized with adaptive *V_bl_* compensation by mitigating the interference from WLn+1.

According to (18), the NWI decreases as $V_{blc}$ increases. As shown in Figure 7a, the potential barrier of the victim cell drops significantly as $V_{bl}$ increases. The $V_{t}$ read from the $I_{d}$-$V_{g}$ curve also decreases as shown in the inset. Therefore, any NWI value caused by different program-state combinations can be compensated by a specific increase of $V_{blc}$. In the following parts, the TCAD simulations are performed to validate this mitigation countermeasure. 

In the actual 3D NAND TLC program, each cell could be written as a state of Erase, A, B, C, D, E, F, and G. From the TCAD results in Figure 7b, we can see that, when interfered by the higher WLn+1 state, the NWI curve of WLn moves upward. A higher WLn+1 state means a larger number of trapped electrons are programmed, which leads to stronger channel potential interference and a larger shift on $V_{t}$ of WLn. Meanwhile, in each curve, the higher WLn state has a smaller $V_{t}$ shift.

When $V_{bl}$ increases from 0.50 V to 0.53 V, the NWI curves of all-state combinations in Figure 7c show an overall downward shift, which indicates that increasing $V_{bl}$ significantly reduces NWI. However, if the same $V_{blc}$ is applied to all points, only a few combinations can ideally have an NWI reduced to zero, whereas other combinations are still in high NWI or are overcompensated.

Therefore, NWI caused by different WLn+1 states should be compensated with appropriate $V_{bl}$. By collecting the $V_{bl}$ values that make the lowest point of each curve in Figure 7c zero and repeating the simulations, the adaptive $V_{bl}$ values required for eight WLn+1 states are shown in the label of Figure 7d. In the second reading of WLn cells, different adaptive $V_{bl}$ values are applied to BLs in terms of WLn+1 states. The simulation results are shown in Figure 7d. Compared with the single $V_{blc}$ in Figure 7c, this adaptive scheme can ensure that the NWI is sufficiently reduced for all combinations without overcompensation and can achieve balanced compensation for all points. Compared with the conventional read scheme, the proposed adaptive $V_{bl}$ countermeasure adds a pre-read phase of WLn+1 before reading WLn. After detecting the WLn+1 state, we raised the $V_{bl}$ to the corresponding adaptive value when reading WLn.

Furthermore, the increased $V_{bl}$ is transmitted through the opened channel and enhances the local DIBL effect at the virtual drain of the read cell, which is the mechanistic reason why the adaptive $V_{bl}$ scheme can mitigate NWI. Specifically, higher $V_{bl}$ causes the channel potential near the virtual drain of WLn to rise. In terms of the local DIBL principle, as the virtual drain potential increases, the electronic reading barrier in the short channel will correspondingly decrease, resulting in a lower read $V_{t}$. As a result, the local potential reduction caused by the NWI effect can be mitigated by the adaptive $V_{bl}$ scheme.

### 5.2. Degressive Adaptive V_bl_ Countermeasure

In Figure 7d, the lower WLn state still suffers from greater residual NWI at a certain WLn+1 state. To avoid $V_{bl}$ overcompensation, each curve has only one adaptive $V_{bl}$ value to make its minimum NWI point (WLn G state) zero. Thus, some residual NWI still exists on other WLn states, and the degree of interference is different.

**Figure 8:**
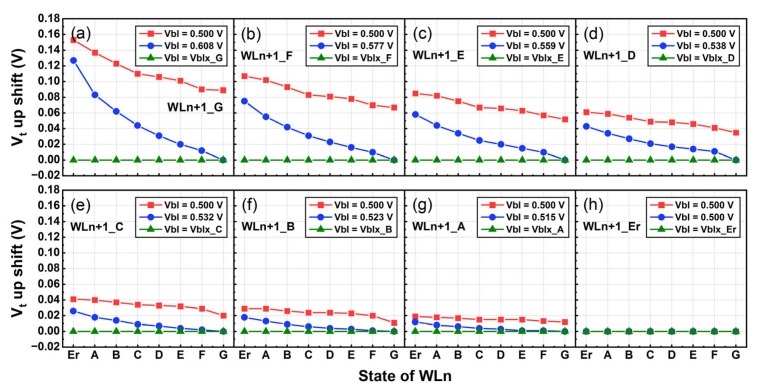
The NWI curves of WLn interfered by WLn+1 (**a**) G, (**b**) F, (**c**) E, (**d**) D, (**e**) C, (**f**) B, (**g**) A, and (**h**) Erase states. The red curve represents original NWI. The blue curve is the NWI result only with adaptive V_bl_ in terms of pre-reading WLn+1 states. The green curve is the NWI result with degressive adaptive V_bl_ according to the pre-reading of WLn+1 states and the read sequence of WLn states.

Here a degressive adaptive $V_{bl}$ countermeasure is further developed. It can eliminate residual NWI differences caused by other WLn states, so as to realize all-state equilibrium compensation. Under the interference of a certain WLn+1 state, the $V_{bl}$ should be increased again based on the adaptive $V_{bl}$ value that is used for each curve above, aiming to reduce the residual NWI. This scheme has been successfully tested by TCAD simulations. As shown in the Figure 8a–h, which show the NWI curves of WLn interfered by the respective G–Erase states of WLn+1. In each figure, from the G to Erase states and based on the adaptive $V_{bl}$ value before, each state is increased as a new series of adaptive $V_{bl}$ values, so that the overall curve decreases towards the X(NWI) = 0 (where no NWI is caused by WLn+1 Erase state, thus maintaining the original $V_{bl}$). There are 64 state points combined with the WLn Erase–G states and the WLn+1 Erase–G states, and each point has a corresponding new adaptive $V_{bl}$, which can make the NWI values approach 0. Read verify is generally from Erase to G states, thus the adaptive $V_{bl}$ in this scheme shows a degressive sequence. The degressive adaptive $V_{bl}$ values measured by TCAD simulation tests are shown in Table 2. The horizontal row represents the $V_{bl}$ adapted according to the states pre-read from WLn+1. The vertical column represents the $V_{bl}$ adapted according to the verification order of TLC levels when reading WLn. Exactly, the adaptive $V_{bl}$ is degressive as the WLn state decreases.

This scheme requires $V_{bl}$ adjustment based on the WLn and WLn+1 states. The WLn+1 state can be detected in advance by the pre-read operation, but the WLn state cannot. After pre-reading the WLn+1 states, corresponding degressive $V_{bl}$ values can be measured as shown in the columns of Table 2. The actual 3D NAND device uses the step verify voltage to read the WLn state. Each state corresponds to a unique $V_{verify,read}$ step voltage, and an exactly degressive adaptive $V_{bl}$ is a certain value for each state. This means that the degressive adaptive $V_{bl}$ can be matched with $V_{verify,read}$. Therefore, with the $V_{verify,read}$ sensing time for different state levels, it is feasible to increase the initial $V_{bl}$ to a specific degressive adaptive value when reading WLn. Thus, the $V_{bl}$ becomes the $V_{bl,read}$ step voltage so that the degressive adaptive $V_{bl}$ scheme can be implemented to optimize all NWI states.

### 5.3. Three-Dimensional NAND Chip Test and Verify

**Figure 9:**
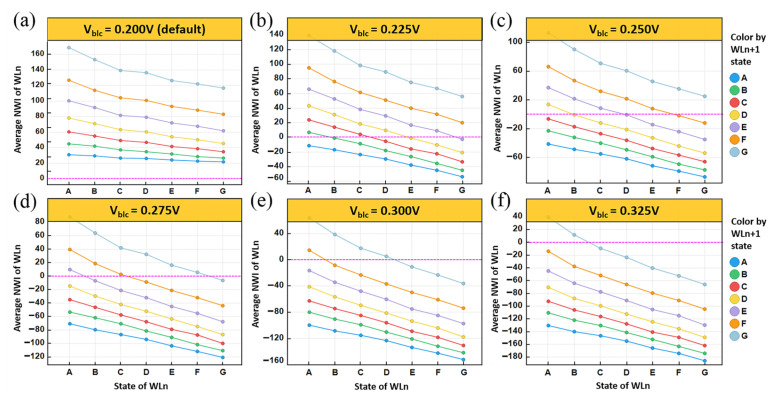
All-state NWI curves drawn from chip test with different V_blc_ are shown in (**a**–**f**). NWI has a dependency on the V_blc_ during reading. A higher V_blc_ applied during reading makes less NWI than the default.

**Figure 10:**
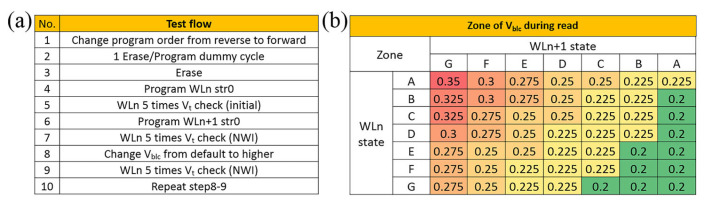
(**a**) Test flow of a 3D NAND chip for NWI. Program string (str0) randomly to collect all state combinations of WLn and WLn+1. (**b**) A set of V_bl_ compensation for all states collected from chip tests. Similar to Table 2, each V_bl_ is the value that minimizes the NWI of a specific state combination. To simplify operations, zones in the same color are victim situations with similar NWI, so these can share the same V_blc_.

A 32Gb TLC NAND flash chip was tested to confirm the adaptive $V_{bl}$ countermeasure proposed above. The chip was tested for NWI effect on the NAND analyzer test platform. All-state NWI curves drawn from chip test with different $V_{blc}$ are shown in the Figure 9a–f. Similar to the TCAD simulations, the chip test adopted the normal order program sequence. At 25 degrees Celsius, the experiment was carried out according to the test flow as shown in the Figure 10a. By changing the default $V_{bl}$ to higher $V_{blc}$ and repeating test flow steps 8–9, several all-state NWI curves with different $V_{blc}$ (partial data) were obtained, as shown in Figure 9. These NWI values were calculated from the difference in $V_{t}$ read between steps 5 and 9 and clearly show that varying the increment of $V_{bl}$ can significantly mitigate the $V_{t}$ upward shift caused by NWI.

With the enhancement of $V_{blc}$, the entire NWI curve gradually moves downward, and each point of the curve alternately sweeps the NWI = 0 axis, as shown in Figure 9. This indicates that for each point affected by the combination of the WLn state and the WLn+1 state, there is always a corresponding $V_{blc}$ that can make its NWI close to zero. By collecting the $V_{blc}$ values that satisfy this condition in the NAND test, a set of $V_{blc}$ compensations for all states is listed in Figure 10b. This result indicates that the degressive adaptive $V_{bl}$ countermeasure can uniformly and effectively minimize the NWI effect on all states of 3D NAND cells. In actual chip operation, the states with similar NWI can be classified into one zone, and the same zone will use the same $V_{bl}$ for compensation. This can shorten the compensation time and simultaneously minimize the NWI below as tolerable level as possible to avoid data errors. The NAND test results confirm the rational adaptive $V_{bl}$ theory and the feasible $V_{bl}$ operation scheme, which shows the potential to alleviate kindred NWI problems on a 3D NAND flash.

## 6. Discussion and Analysis

In this section, we present the discussion about the proposed NWI physical device model and adaptive $V_{bl}$ scheme, in terms of further analysis and future research directions.

### 6.1. NWI Physical Device Model and Channel Potential Analysis

In this study, a multi-parameter physical device model is proposed based on the actual performance of NWI in 3D NAND flash. The model is used to reflect the influence of each parameter on NWI and their interaction. Among these,$\;I_{sense}$, $V_{read}$, $V_{bl}$, $V_{t,parasitic},\;V_{t,n+1}$, and the material/structure parameters of the device are the main influencing factors of NWI. The model shows the compositional structure of NWI and provides an explanation of each parameter, which is essential for further investigation of the NWI mechanism. $I_{sense}$, as the channel current of the read cell, directly reflects the threshold voltage magnitude of WLn. Contrary to the previous view that NWI is mainly caused by redundant electrons, this suggests that the storage state of the affected cell plays a crucial role in determining the magnitude of NWI. Within the appropriate range, a slightly lower $I_{sense}$ and a higher storage state are beneficial for reducing NWI. $V_{read}$ as read pass voltage, especially applied on WLn+1 and WLn-1, has a significant effect on the NWI of WLn. In the channel potential analysis of NWI, it was found that $V_{read}$ can directly compensate the potential loss of WLn due to the potential superposition and the local DIBL effect caused by the short channel. The large potential gradient near WLn puts it in a state where it is susceptible to interference from the neighbor potential variations. The difference of $V_{read}$ applied on WLn+1 between the two reads of WLn can be increased while satisfying the normal conduction of the channel under all other WL cells, so that the channel potential barrier of WLn is at the same level prior to and after all the upper cells are programmed. Through chip tests and parameter training, properly raising the adjacent $V_{read}$ can avoid causing derived program disturbances and read disturbances. The $V_{bl}$ bitline voltage is used as a virtual drain voltage in the WLn read phase, and the voltage can directly affect the data read results through the transfer of the passed channel. However, this transfer will be inevitably weakened by all of the upper programmed cells on the drain side; however, this loss is only slight due to the presence of the read pass voltage. This weakening is most pronounced only near WLn+1, as at the location of $V_{read}$ it is not possible to fully conduct the channel. $V_{t,parasitic}$ is defined as the equivalent threshold voltage of electrons between cells, which represents the fraction of electrons in the wordline isolation layer caused by diffusion or drift, that still have a contribution to the threshold voltage of adjacent cells. This fraction of the electrons is difficult to avoid, but its number can be reduced by some novel program methods to cover the effects or by material and process blocking. $V_{t,n+1}$ is the state in which the WLn+1 cell is programmed and also one of the reasons why WLn suffers from differential NWI. The structural parameters of the device mostly depend on the device process and technology nodes. In particular, the channel length is critical for the generation of NWI effect. As the device size continues to scale, further reductions in the cell channel length may cause even more cells to have a large NWI on WLn, exacerbating the data shift. Materials for flash memory devices have more research options and by improving the process or by using advanced dielectric and charge trapping materials, the diffusion and drift of trapped electrons in the CTL can be reduced, with lower charge loss and NWI.

The channel potential analysis method based on the potential superposition and local DIBL effect was described in detail in Section 4. This section serves as an extension of the physical device model at the channel potential level, since it can be seen that the key parameters in the physical device model mostly related to the channel potential. The physical device model adopts a more intuitive description method for the convenience of the test developer’s quick judgment. The channel potential analysis serves as the mechanism for NWI generation. It uses the local DIBL effect to essentially explain that the source of the NWI is the virtual drain potential loss in the adjacent channel. On the other hand, both the physical device model and the channel potential analysis provide a theoretical basis for the subsequently proposed adaptive $V_{bl}$ scheme, which guides $V_{bl}$ to modulate the channel potential and thus reduce NWI.

In terms of benefits, the NWI physical device model is the first physical model to reflect the NWI effect in BiCS 3D NAND flash memory, which is one of the latest technologies in industrial 3D NAND flash devices. In fact, the doping structure of 2D NAND devices is very different from the virtual source/drain structure of 3D NAND. Therefore, the proposed physical model is an update based on the latest 3D NAND devices. In addition, the structure of the NWI physical device model is composed of the actual EPR parameters, which is more convenient for reflecting the parameter influence on the NWI results. Thus, the model serves as a better reference in the application of real chip operations. With more adjustable electrical parameters, this model can help device testers and designers to evaluate NWI noise faster and more accurately in the field. Most importantly, the NWI physical device model highlights the use of $I_{sense}$, $V_{read}$, $V_{bl}$, $V_{t,parasitic}$, and $V_{t,n+1}$, providing theoretical support not only for the adaptive $V_{bl}$ scheme, but also for many other optimization strategies. Of course, in terms of possible drawbacks, this model is an ideal model based on the standard MONOS flash cell and is mostly used for the qualitative analysis of NWI patterns. An accurate quantitative analysis of NWI results by the ideal model is not the best choice. In practice, NWI results are more often obtained by NAND platform testing and then backwards extrapolation. In addition, the model is proposed for NWI problems, so its application to other reliability problems such as data retention or process defect interference also remains to be investigated.

In the practical chip test, different patterns can be adopted according to different application scenarios and performance requirements. After the basic parameter training is completed, new suitable compensation parameters can be obtained by testing the corresponding pattern for the NWI environment. The identification of influential adjustable parameters and cell states is essential for further exploration of the NWI mechanism. The NWI physical device model integrates various relevant device factors, reflecting the interaction of each factor and its contribution to NWI. It also provides theoretical support for the development of appropriate optimization schemes. The exploration of potential factors and novel structures for NWI will continue.

### 6.2. Analysis of Adaptive V_bl_ Countermeasure

In this paper, a scheme utilizing adaptive bitline voltage regulation for NWI in the read phase is proposed and verified by 3D NAND chip test. The scheme is supported by the physical device model and the channel potential theory. The channel potential superposition and the local DIBL effect caused by NWI will lead to a decrease in the virtual drain potential of the read cell. The bitline voltage $V_{bl}$ can be transferred to the virtual drain of the read cell through the passed channel on the drain side. Therefore, the virtual drain voltage loss can be basically compensated for by a series of adaptive $V_{bl}$ values in the read phase. This scheme has a small increase in $V_{bl}$, and is only implemented in the read phase after all programming is completed, with minimal impact on other operations. This scheme requires a quick pre-read of the programmed WLn+1 in the read phase, which is used to determine the state of WLn+1. 

In fact, as shown in Figure 10b, the NWI differences between some adjacent state combinations are not significant. In such cases, within the tolerance range, the same $V_{bl}$ can be applied to these regions, which can reduce the frequency of voltage changes and improve the efficiency of this scheme. Therefore, for pre-reading WLn+1, the states with similar $V_{bl}$ compensation values can be grouped into the same zone. If only A to D, E to F, and G states are considered as three zones, two read voltage levels can be used to distinguish between the low, medium, and high states of WLn+1. If the grouping is reduced to two zones, only one read voltage level is required, which can shorten the time to achieve fast reading. Similarly, for reading WLn, the eight states of TLC can also be divided into several zones based on the NWI proximity. For instance, if the A to D and E to G states are divided into high and low zones, then in a normal read verify phase, only two adaptive $V_{bl}$ values need to be used in the corresponding high (A–D) and low (E–G) verification zones. During a single normal read verification process, only two adaptive $V_{bl}$ values need to be used in the corresponding high and low state verification intervals. A reasonable partition can greatly improve the implementation efficiency of this scheme.

On the other hand, the NWI variations caused by the stacking of upper wordlines can be overcome by the cooperation of $V_{bl}$ and $V_{read}$. In today’s 3D NAND flash technology, the stacking of wordline layers (>200 layers) may lead to a non-negligible accumulation of channel resistance after all cells have been programmed. To address this issue without introducing additional noise, appropriate compensation for $V_{read}$ can be achieved by subsequent pattern tests. 

In addition, even if the random combination of upper programmed WLs still causes channel resistance variations among different strings (which can be understood as differences in virtual drain potential loss of WLn), the adaptive $V_{bl}$ scheme can also cover this effect. This is because the pre-read result of WLn+1 in the adaptive $V_{bl}$ scheme can actually reflect the channel resistance difference. If the virtual drain potential of WLn shows string differences due to the stacking of upper WLs, the pre-read $V_{t}$ of WLn+1 will also show differences. Depending on the $V_{t}$ result of WLn+1, the $V_{bl}$ compensation will also change, even jumping to another zone to cover this effect. The optimization accuracy of such difference depends on the fault tolerance of the NAND flash memory system. As mentioned above, this scheme can minimize such interference with a balance between efficiency and compensation accuracy.

The study focuses on exploring strategies to improve NWI by using adaptive $V_{bl}$. Physical device model and channel potential analysis also provide directions, such as $V_{read}$/$I_{sense}$ electrical parameter adjustment schemes, novel program schemes based on cell state combinations, and advanced device structures and materials. These studies will help us further investigate the set of NWI issues and provide more reliable data storage performance for NAND flash-based sensor systems.

## 7. Conclusions

In this study, to alleviate data disturbances and NWI effects in a 3D NAND flash, a physical device model was established and an adaptive $V_{bl}$ countermeasure was proposed, with the NWI mechanism analysis based on channel potential. First, in TCAD, it was found that the channel potential change under read bias condition could reflect the actual NWI more accurately than the floating case. Thus, an NWI device model composed of flash operation parameters was built, which could intuitively suggest the contribution of $V_{read}$, $V_{bl}$, $I_{sense}$ and concerned $V_{t}\;$ to NWI. NWI is composed of two main processes: channel potential superposition and local DIBL effect. Then, it was demonstrated that the local DIBL effect is the main cause of NWI by TCAD simulations with wordline gaps. Finally, an adaptive $V_{bl}$ countermeasure was proposed to mitigate NWI in 3D NAND arrays. In this way, the $V_{bl}$ is boosted in stages according to the combinations of WLn and WLn+1 states, which in turn significantly balances and minimizes the NWI of TLC cells. These patterns were successfully verified in both TCAD and 3D NAND chip test. This study will help to advance NWI optimization and further support efficient prediction of data reliability in 3D NAND flash memory-based sensor systems.

## Figures and Tables

**Table 1:** $V_{t}$ variation and NWI with three different WL gaps.

Gap/nm	*V_t_* (Initial)/V	*V_t_* (NWI)/V	*V_t_* (Shift)/V
20	5.309	5.407	0.098
40	5.310	5.320	0.010
60	5.311	5.311	0.000

**Table 2:** The degressive adaptive V_bl_ values measured by TCAD simulations.

Adaptive V_bl_ (V)		WLn+1 state							
		G	F	E	D	C	B	A	Erase
**WLn state**	**Erase**	2.610	0.769	0.693	0.629	0.584	0.558	0.537	0.500
	**A**	0.854	0.672	0.630	0.590	0.559	0.541	0.526	0.500
	**B**	0.738	0.642	0.609	0.576	0.551	0.535	0.523	0.500
	**C**	0.688	0.623	0.595	0.566	0.544	0.531	0.520	0.500
	**D**	0.656	0.608	0.584	0.560	0.540	0.528	0.518	0.500
	**E**	0.636	0.598	0.577	0.555	0.537	0.526	0.517	0.500
	**F**	0.625	0.591	0.572	0.551	0.535	0.525	0.516	0.500
	**G**	0.608	0.577	0.559	0.538	0.532	0.523	0.515	0.500

The magnitude of the adaptive values can be visually distinguished by the color gra-dation in the Table 2. As the color deepens to red, it represents the cell suffering from more severe NWI and the compensation needs to be greater.

## Data Availability

Data for this research are available upon request via correspondence with the author.
